# Inhibitory interneurons in visual cortical plasticity

**DOI:** 10.1007/s00018-016-2264-4

**Published:** 2016-05-18

**Authors:** Daniëlle van Versendaal, Christiaan N. Levelt

**Affiliations:** 1 Department of Molecular Visual Plasticity, Netherlands Institute for Neuroscience, Institute of the Royal Netherlands Academy of Arts and Sciences, Meibergdreef 47, 1105 BA Amsterdam, The Netherlands; 2 Department of Molecular and Cellular Neurobiology, Center for Neurogenomics and Cognitive Research, VU University Amsterdam, de Boelelaan 1085, 1081HV Amsterdam, The Netherlands

**Keywords:** Ocular dominance plasticity, Adult, Perceptual learning, Inhibition V1, Somatostatin, Vasoactive intestinal peptide, Parvalbumin, Neurogliaform cells

## Abstract

For proper maturation of the neocortex and acquisition of specific functions and skills, exposure to sensory stimuli is vital during critical periods of development when synaptic connectivity is highly malleable. To preserve reliable cortical processing, it is essential that these critical periods end after which learning becomes more conditional and active interaction with the environment becomes more important. How these age-dependent forms of plasticity are regulated has been studied extensively in the primary visual cortex. This has revealed that inhibitory innervation plays a crucial role and that a temporary decrease in inhibition is essential for plasticity to take place. Here, we discuss how different interneuron subsets regulate plasticity during different stages of cortical maturation. We propose a theory in which different interneuron subsets select the sources of neuronal input that undergo plasticity.

## The regulation of cortical plasticity

The brain shows a tremendous ability to adapt to its ever-changing environment. The root of this adaptation is the formation and refinement of neural circuits, allowing our brains to develop, acquire knowledge, learn new skills, and recover from injuries. The way experience influences the structure and function of neuronal connections, referred to as experience-dependent plasticity, and changes during the course of our lives.

During early development, passive exposure to input from the environment is important for proper maturation of the neocortex. In fact, for acquiring and retaining certain functions and skills, it is an absolute requirement that such exposure takes place during well-defined periods of development. These periods during which neural connectivity is especially malleable are called “critical periods.” Critical periods were first formally defined by Austrian biologist Konrad Lorenz who discovered that the first hours after hatching are crucial for graylag geese to bond with their mother [1]. In humans, the presence of critical periods in speech development is demonstrated by rare cases of so-called feral children who grow up isolated from human contact. Not being exposed to language vocalizations interferes with their ability to perceive and produce phonemes, the building blocks of language [2, 3]. A situation, which applies to all of us, is that if we are not exposed to the sounds of a particular language during the first years after birth, our auditory system has great difficulty distinguishing particular language-specific sounds [2, 3]. A proper development of the visual cortex also requires experience. This is exemplified by amblyopia (or “lazy eye”) in which low-quality input from one eye for an extended period of time causes its inputs to the cortex to become less effective, leading to lowered cortical acuity and reduced depth perception [4]. Its treatment, correction of the primary visual deficit in the affected eye and temporary occlusion of the dominant eye, has to occur before the age of 8 years when the critical period for this form of plasticity closes [5].

From these examples, it is clear that limited or erroneous experience during critical periods has lifelong consequences. This raises the question why critical periods close at all. Would it not be better if high levels of plasticity were retained throughout life? For several reasons, it is important for critical periods to end. First, while high plasticity levels improve function based on experience, they also cause vulnerability to deterioration of optimal function induced by incongruous inputs. Second, lower and higher brain regions are connected through feedforward and feedback connections [6, 7]. If the lower cortical areas continuously change the way they process information, the bidirectional communication with higher cortical areas would be severely hampered.

Although critical periods close at a particular age, a certain level of plasticity is retained, albeit of a different nature. First, learning becomes more conditional and often requires instructions. The passive exposure to stimuli is much less efficient in driving plasticity, and active interaction with the environment involving various forms of reinforcement becomes the dominant way of learning. Second, the substrate of plasticity changes. During critical periods, feedforward connections undergo extensive changes [8], while later in life, associative inputs are the more malleable [9, 10].

How is this switch from critical period to adult forms of plasticity achieved? It has been known for quite some time now that the development of inhibitory innervation plays a crucial role in opening and closing critical periods [11–13]. More recently, evidence is accumulating, which suggests that different subsets of inhibitory interneurons regulate plasticity levels during critical periods and in adulthood [14, 15]. They may contribute to selecting different sources of neuronal input and regulate, which inputs undergo plasticity under specific circumstances. Here, we review the properties of different interneuron subsets and propose a hypothesis on how they may regulate different forms of plasticity during development of the visual cortex.

## Plasticity at different stages of cortical development

The primary visual cortex (V1) has been used extensively to study cortical plasticity during development and adulthood. During the first weeks after mice are born, plasticity in V1 is driven by spontaneous activity originating in the thalamus and cortex [16], and later also by spontaneous retinal activity [17]. This spontaneous-activity-mediated plasticity is essential for setting up the thalamocortical and cortical circuitry of the visual system. Later on, visual input from the two eyes starts to drive plasticity and refines these circuits in V1. The most studied form of plasticity during this developmental stage is ocular dominance (OD) plasticity [18], which is important for the development of binocular vision and when misguided, can cause amblyopia. It can be induced experimentally by temporarily closing one eye. Visual responses of the two retinas are propagated to the visual system through the optic nerves [19]. These partially cross at the optical chiasm and project to the lateral geniculate nuclei (LGN) in the thalamus of both hemispheres. Visual information from LGN is relayed predominantly to layer 4 of the primary visual cortex (V1). Occlusion of one eye [monocular deprivation (MD)] during the critical period shifts the responsiveness of neurons to input from the non-deprived eye [20, 21]. This functional shift in OD is accompanied by extensive rearrangements of thalamocortical projections, with those serving the closed eye retracting and those of the open eye expanding [8, 22–24]. In addition to dendritic spines, the protrusions on excitatory neurons on which most excitatory synapses are located show structural plasticity during OD plasticity [25]. Making use of in vivo two-photon microscopy in mice in which a fraction of cortical neurons are expressing a green fluorescent protein (GFP), it was found that MD causes a rapid increase in the loss and gain of dendritic spines of layer 2/3 and 5 pyramidal neurons in V1 [25, 26].

In the first months after critical period closure, OD plasticity can still be induced but less efficiently so and does not involve rearrangement of thalamocortical projections [23, 27, 28]. While MD still increases spine turnover in pyramidal neurons in layer 5, this is no longer the case in layer 2/3 [29]. After critical period closure, additional forms of plasticity become more dominant in V1, most importantly perceptual learning. This is the improvement in the ability to detect or discriminate visual stimuli induced by repeated practice. Perceptual learning is the type of learning that allows experienced birdwatchers to spot the bird in a tree which an untrained person would overlook. It involves various visual cortical areas, including V1 [30–34], and is strongly influenced by reinforcement signals, such as reward or punishment [35]. Perceptual learning often requires the interaction of feedforward information with contextual information. Such contextual information is provided to V1 by feedback connections. These connections are, therefore, likely to be an important substrate of plasticity during perceptual learning instead of the feedforward connections that are fine-tuned during the critical period.

## Plasticity regulation

How the critical period of OD plasticity is opened and closed and the transition to adult forms of plasticity are achieved is under intense investigation. The strong decline in structural plasticity after critical period closure suggests that cell-intrinsic mechanisms restricting structural plasticity are responsible for critical period closure. Indeed, inactivating certain signaling pathways, which inhibit structural plasticity, interferes with critical period closure [36–39]. However, inhibitory innervation has been found to be at least as important in this process and to represent a reversible and specific regulator of plasticity levels in the developing and adult cortex [40–42]. Mice deficient for one of the two isoforms of glutamatergic acid decarboxylase (GAD65), a γ-aminobutyric acid (GABA) synthesizing enzyme, have reduced GABA release and show no OD plasticity. This can be rescued by increasing inhibition pharmacologically with diazepam [11]. Furthermore, increasing the level of GABAergic transmission by benzodiazepine infusion in very young mice promotes the early onset of the critical period of OD plasticity [12]. A precocious critical period can also be induced by accelerating the maturation of inhibitory innervation. This can be achieved by genetically increasing cortical BDNF levels or enzymatic removal of polysialic acid, which is mostly associated with neural cell adhesion molecules [13, 43, 44]. The maturation of inhibitory innervation is thus an important factor in critical period onset. Further increasing inhibition during development closes the critical period [13, 44–47].

These discoveries suggest that a gradual increase in inhibition defines the critical period. However, it was recently discovered that a temporary suppression of inhibition occurs during plasticity in the visual cortex, both during the critical period [47], and adulthood [14, 48–50] and increases plasticity levels [47]. Depending on the age or behavioral state during which plasticity is induced and what the substrates of cortical plasticity are, this disinhibition involves different interneuron subsets and underlying mechanisms. To understand how this may work, knowledge on the various cortical interneuron subsets and their connectivity and functions is essential.

## The main interneuron subtypes

**Fig. 1:**
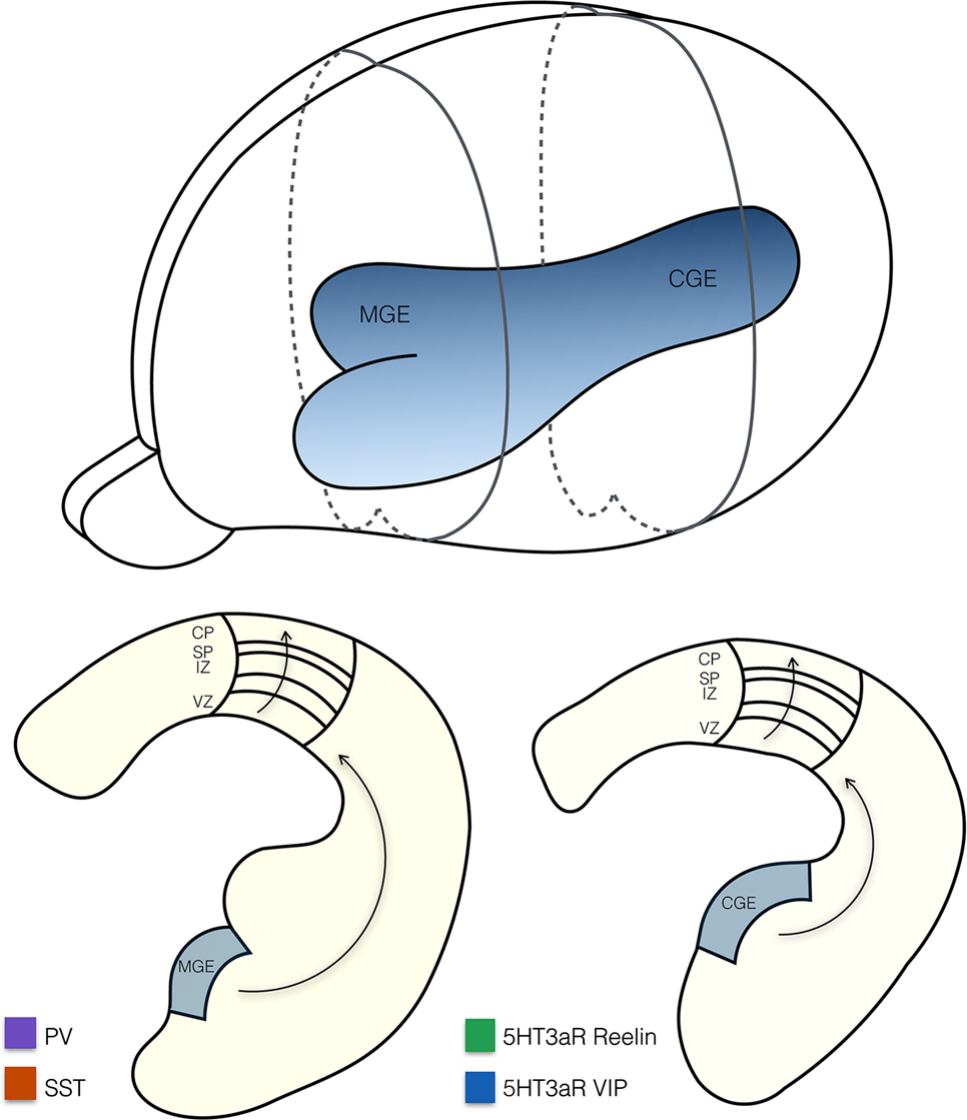
Anatomy of the embryonic telencephalon showing the two main structures from which inhibitory interneurons are derived: the medial ganglionic eminence (MGE) and the caudal ganglionic eminence (CGE), as a 3D structure in the intact brain as well as in two sections. The MGE and CGE give rise to different interneuron subtypes: 5HT3aR expressing interneurons are derived from the CGE and PV and SST expressing interneurons are derived from the MGE. Progenitor cells tangentially migrate to the appropriate cortical area before they radially position themselves via the ventricular zone (VZ), intermediate zone (IZ) and subplate (SP) to their final laminar position in the cortical plate (CP)

GABAergic interneurons make up for only 10–20 % of the neuronal population in the cortex, yet their function is vital for shaping cortical activity. The high diversity of interneuron subsets in terms of gene expression profiles, physiological properties, and connectivity patterns is reflected in their specialized functional roles in cortical processing, such as balancing network activity, tuning width sharpening, and controlling the flow of information and synchronization at the circuit level [51, 52]. In recent years, we obtained a much better understanding of the developmental origins, genetic factors, and activity-dependent events that shape interneuron development and differentiation. In contrast to excitatory pyramidal cells, which originate from the subventricular zone lining the developing cortex, inhibitory interneurons are derived from a more distant source: the ganglionic eminences in the ventral portion of the telencephalon [53] (Fig. 1). In mice, cortical interneurons are first generated within the medial ganglionic eminence (MGE) with a peak production at around embryonic day 14 (E14), followed by the interneurons that are derived from the caudal ganglionic eminence (CGE) around E16 [54, 55]. Notably, different interneuron subtypes are generated within the MGE and CGE (Fig. 1).

**Fig. 2:**
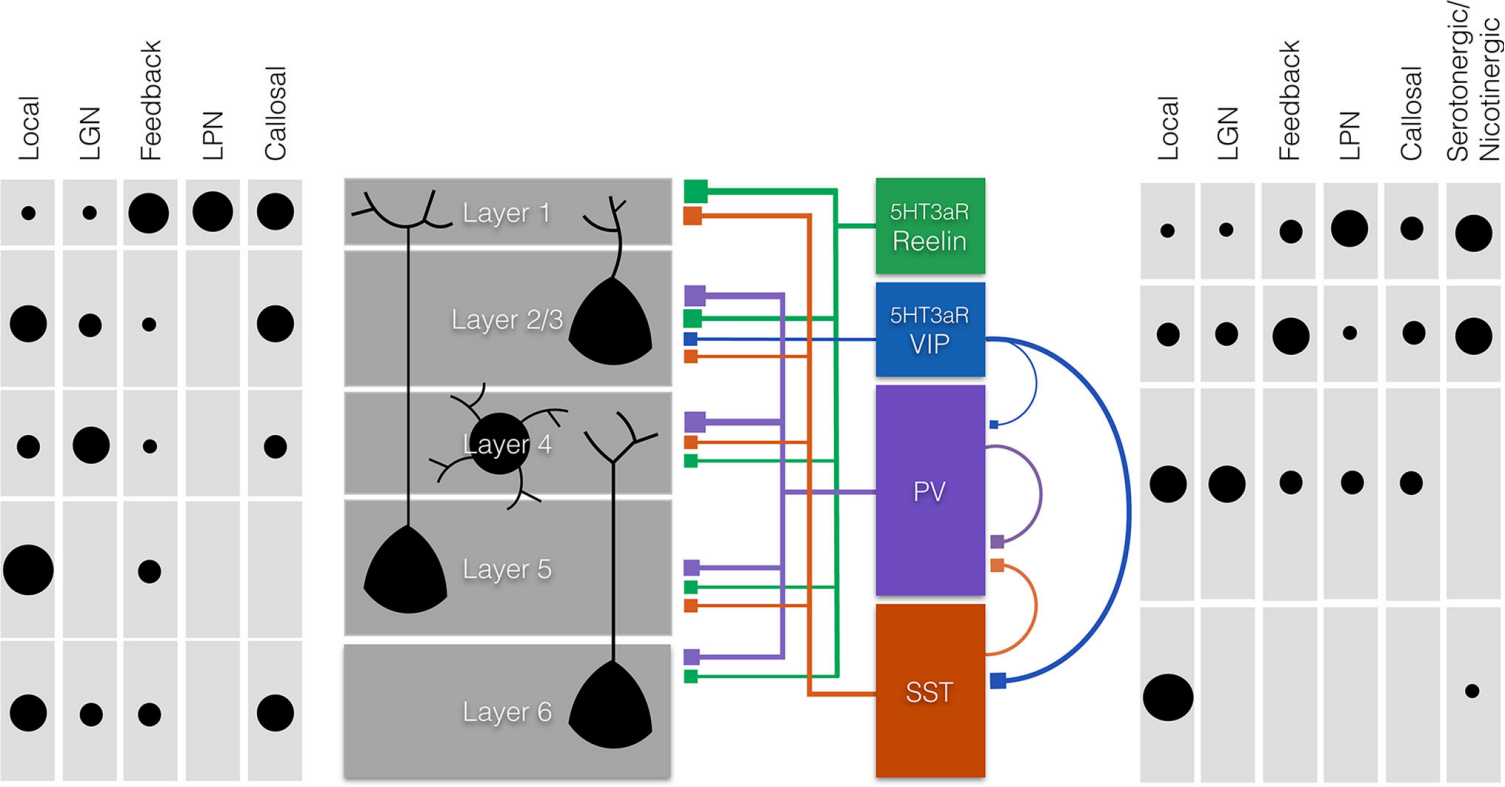
Schematic representation of the main projections to and from pyramidal cells and interneurons within the six layers of the primary visual cortex (V1). Shown are rough estimates of densities (*black circles*) from local, thalamic (lateral geniculate nucleus (LGN) and the lateral posterior nucleus (LPN) of the thalamus), feedback and callosal projections to the different layers of V1 (*left panel*) and to different subtypes of interneurons (*right panel*). Estimates are based on the literature and Allen Mouse Brain Connectivity Atlas [158]. Layers 5 and 2/3 mainly receive local inputs, whereas layer 4 mostly receives thalamic input from the LGN. Conversely, layer 1 mostly receives thalamic input from the LPN, callosal inputs, and feedback projections. The subtypes of interneurons discussed in this article (*middle right panel*) express either a combination of the serotonin receptor 5HT3aR with reelin or vasoactive intestinal peptide (VIP) or are positive for parvalbumin (PV) or somatostatin (SST). Neurogliaform cells (NGF) express 5HT3aR and reelin and are indicated in *green*, 5HT3aR positive interneurons that express VIP are indicated in *blue*, chandelier and basket cells express PV and are indicated in *purple,* and finally, Martinotti cells that express SST are indicated in *red*. Both NGF cells and VIP+ interneurons are strongly responsive to nicotinergic and serotonergic neuromodulatory inputs and inputs from higher brain regions (feedback and callosal). NGF cells provide strong local inhibition through volume release of GABA mainly in the upper layers, but also in deeper layers. They inhibit all types of local excitatory and inhibitory neurons (not shown in figure). VIP interneurons mainly innervate other interneurons (SST+ and to a lesser extent PV+ interneurons). Basket cells are mainly innervated by thalamic (LGN) and local excitatory axons. They innervate the proximal dendrites and somata of pyramidal cells with a bias to layer 2/3 and layer 4. They receive inhibitory inputs from SST+ and VIP+ interneurons and other basket cells. Chandelier cells are special in the sense that they form inhibitory synapses on the axon initial segment of pyramidal cells (not shown in figure). Finally, Martinotti cells predominantly receive local inputs and preferentially form inhibitory synapses on distal dendrites and tufts of pyramidal cells

Around birth, postmitotic interneuron progenitors migrate tangentially to the appropriate cortical area before they migrate radially via the ventricular zone (VZ), intermediate zone (IZ), and subplate (SP) to their final laminar position in the cortical plate (CP) [56, 57], (Fig. 1). MGE-derived interneurons populate the cortical layers in an inside-out order as do pyramidal cells. CGE-derived interneurons do not follow this sequence and accumulate predominantly in the top layers [54, 57]. During the first postnatal week, the progenitor cells specify into different subclasses of interneurons during which they acquire their mature morphologies, neurochemical expression patterns, and electrical properties, and form stereotypical cortical circuits [52]. Here, we focus on four interneuron subtypes that make up for the majority of cortical interneurons: two MGE-derived subtypes that express either the Ca^2+^ binding protein parvalbumin (PV) or the neuropeptides somatostatin (SST), and two CGE-derived subtypes both expressing the serotonin receptor 5HT3aR together with either vasoactive intestinal peptide (VIP), or reelin [58–60] (Fig. 2).

### PV-expressing interneurons

Interneurons expressing PV are MGE derived and are the largest group of interneurons in the cortex, accounting for 40 % of the total GABAergic population [55, 58]. Of the PV-expressing interneurons, a small proportion is constituted by the chandelier cells that target the axon initial segments of principle neurons. Evidence suggests that chandelier cells depolarize or hyperpolarize principal cells depending on whether these cells are quiescent or whether their membrane potential fluctuates, as is often observed in vivo [61, 62]. The actual function of chandelier cells is not yet understood.

Most PV+ interneurons are fast-spiking basket cells that predominantly innervate proximal dendrites and somata of their targets, and provide the main source of somatic inhibition [63–65]. Their cell bodies are found in all cortical layers with the exception of layer 1, and they are most numerous in layers 4 and 5 [65–68]. Most basket cells project locally, but in some cases, their axons can cross different layers [58, 66]. Basket cells receive the bulk of the thalamic input to interneurons and are the dominant interneuron subset exerting control of pyramidal cell firing [69–71] (Fig. 2). They, thus, provide strong feedforward inhibition and may gate sensory input from the thalamus.

Fast-spiking basket cells also receive pooled input from local cortical neurons with different tuning properties [72, 73]. This causes them to be only weakly tuned but highly suited for regulating the dynamic range of cortical responses. This is a crucial function in highly recurrent networks. While such networks enable the cortex to selectively amplify relevant information, they carry the risk of runaway activity. Optogenetic approaches have shown that PV+ basket cells reduce the activity of cortical excitatory neurons by both thresholding and scaling their responses, thus keeping the system within its optimal dynamic range [74, 75].

PV+ basket cells are also responsible for ensuring that the timing of sensory stimuli is accurately represented in sensory systems [76]. Cortical neurons summate sensory inputs that occur within a set period of time, thus triggering a response only if they coincide. PV+-basket-cell-mediated feedforward inhibition can narrow this window of integration and effectively regulate temporal summation by rapidly hyperpolarizing the neuron after receiving synaptic input. Finally, PV+ basket cells are also believed to orchestrate oscillatory activity in the gamma range (30–80 Hz) made possible by their fast and non-adapting firing properties and their extensive interconnectivity through inhibitory synapses and gap junctions [77–80].

### SST-expressing interneurons

The second group of MGE-derived interneurons expresses SST and makes up for 30 % of all cortical interneurons [58]. SST+ interneurons are typically Martinotti cells. The somata of these cells are most abundant in layers 2/3 and 5 and excluded from layer 1 [65–67, 81]. SST+ cells receive excitatory input from local pyramidal cells and form most of their inhibitory synapses on the dendritic tufts in layer 1 [65, 81, 82] but also on distal dendrites of neurons in other layers [68]. The distal dendrites mostly receive horizontal connections from other pyramidal neurons situated further away within V1, while the dendritic tufts receive associative and feedback connections from many different cortical areas and thalamic association nuclei, such as pulvinar (lateral posterior nucleus in rodents) (Fig. 2). Inhibitory synapses formed by SST+ interneurons are thus perfectly situated to gate these inputs.

SST+ interneurons are also involved in feature coding, i.e., the sculpting of excitatory neuron responses. A classic example is surround suppression [83]. Neurons in V1 respond most strongly when a visual stimulus of a particular size is presented. When this visual stimulus is enlarged, the neuron will respond more weakly. This results in a relative enhancement of responses to borders of visual stimuli. Surround suppression thus enhances apparent contrast and underlies visual pop-out. This suppression by stimulation of the surrounding area of the classical receptive field in mouse V1 involves suppression by SST+ interneurons with much larger receptive fields [84]. However, SST+ interneuron-mediated inhibition is certainly not the only mechanism responsible for surround suppression, as it is only reduced but not absent under anesthesia when SST+ interneurons have little influence on visual responses in V1 [84–86] or when SST+ interneurons are optogenetically silenced [84].

In contrast to PV+ interneurons, SST+ Martinotti cells do not form inhibitory synapses onto each other, but extensively innervate other interneuron subsets, including PV+ basket cells [87, 88]. A subset of SST+ interneurons in layer 4 exclusively inhibits PV+ interneurons [89]. Activity of SST+ interneurons may, therefore, not only suppress horizontal and feedback connections, but also disinhibit feedforward connections. Finally, some SST+ interneuron subsets have been identified whose functions are not yet understood, including SST+ basket cells throughout the cortex and bitufted cells in layer 2/3 [90].

### VIP-expressing interneurons

The third largest group of interneurons (30 %) expresses the serotonin receptor (5HT3aR); a subset of these also expresses VIP [59]. These VIP+ interneurons are typically bipolar cells that are specialized in inhibiting other interneuron subtypes, especially SST+ interneurons and to a lesser extent PV+ basket cells [87, 91–93]. In addition, VIP+ bitufted cells have been identified, which also inhibit pyramidal neurons [90]. All VIP+ interneurons are activated by cholinergic and serotonergic inputs [94, 95], but also receive long-range intercortical and thalamic inputs (Fig. 2). Suppression of SST+ interneurons by VIP+ interneurons may enhance associative/feedback excitatory inputs. At the same time, it could cause suppression of feedforward connections as suppression of SST+ interneurons disinhibits PV+ interneurons. Because VIP+ interneurons express serotonin and nicotinic acetylcholine receptors, these neuromodulators may thus contribute to switching between feedforward and feedback input to V1 and provide reinforcement signals important for perceptual learning.

### Neurogliaform cells

A second subset of 5HT3aR expressing interneurons consists of the VIP negative, reelin positive neurogliaform (NGF) cells [59, 96]. These interneurons have characteristic spider web morphology and provide inhibition by volume transmission of GABA that acts on postsynaptic targets through the slower metabotropic GABA_B_- and possibly extrasynaptic GABA_A_ receptors [97]. These postsynaptic targets include all excitatory and inhibitory neurons with dendrites in the proximity of the NGF cell. NGF cells also form gap junctions with various other interneuron subsets through which they can synchronize them and exert powerful cortical inhibition [98, 99]. NGF cells can be found mainly in layer 1, where they also express neuron-derived neurotrophic factor (NDNF) [100] but also in layer 2/3 and to a lesser extent in layer 5. NGF cells in layer 2/3 receive thalamic and local cortical input. Layer 1 NGF cells also receive strong callosal and feedback inputs and evidence suggests that they regulate the dendritic integration of feedforward and callosal/feedback inputs [101]. Interestingly, layer 2/3 NGF cells have also been found to inhibit feedforward inhibition by PV+ basket cells [102] showing that like SST+ interneurons, they may have a role in switching between feedforward and feedback inputs.

## Selecting the substrate of plasticity by selective disinhibition

How may this diverse set of interneurons with their various functions work together in regulating plasticity levels during development and in adulthood? We propose that interneurons select the different sources of neuronal input that may be relevant for learning at particular developmental stages and/or under specific circumstances. We will argue that different interneuron subsets are involved in regulating plasticity at the different stages of development: early on, PV+ basket cells are the main regulators of plasticity, while during adulthood, SST+ interneurons, controlled by VIP+ interneurons, appear to become the more dominant regulators of cortical plasticity.

## Regulation of critical period plasticity by PV+ basket cells

Early during development, inhibitory innervation in V1 is weak, and cortical neurons are spontaneously active with high synchronicity [103]. This cortex- and later retina-derived spontaneous activity drives the precise wiring of cortical neurons [16, 104, 105]. It was recently discovered that synapses that exhibit low synchronicity with nearby synapses are more likely to be depressed [104]. This “out-of-sync, lose-your-link” mechanism is believed to underlie the clustering of co-active synapses. The time window for optimal desynchronization-induced plasticity is very broad (1.5–2 s) and matches the duration of spontaneous waves originating in the retina [104]. Once the eyes open, visual inputs start contributing to activity in V1. Based on the precise timing of these inputs, experience-dependent plasticity will optimize cortical neuronal circuits. This improves visual processing leading to increased acuity and fine tunes binocular vision important for depth perception. Thus, plasticity mechanisms must now be adjusted to a new source of information that also has different temporal properties. The development of inhibitory synapses formed by PV+ basket cells is thought to both adjust the temporal aspects of cortical processing and suppress spontaneous activity, thus optimizing conditions for plasticity based on visual input.

## PV+ basket cells suppress spontaneous activity and decrease the window of spike-timing-dependent plasticity

**Fig. 3:**
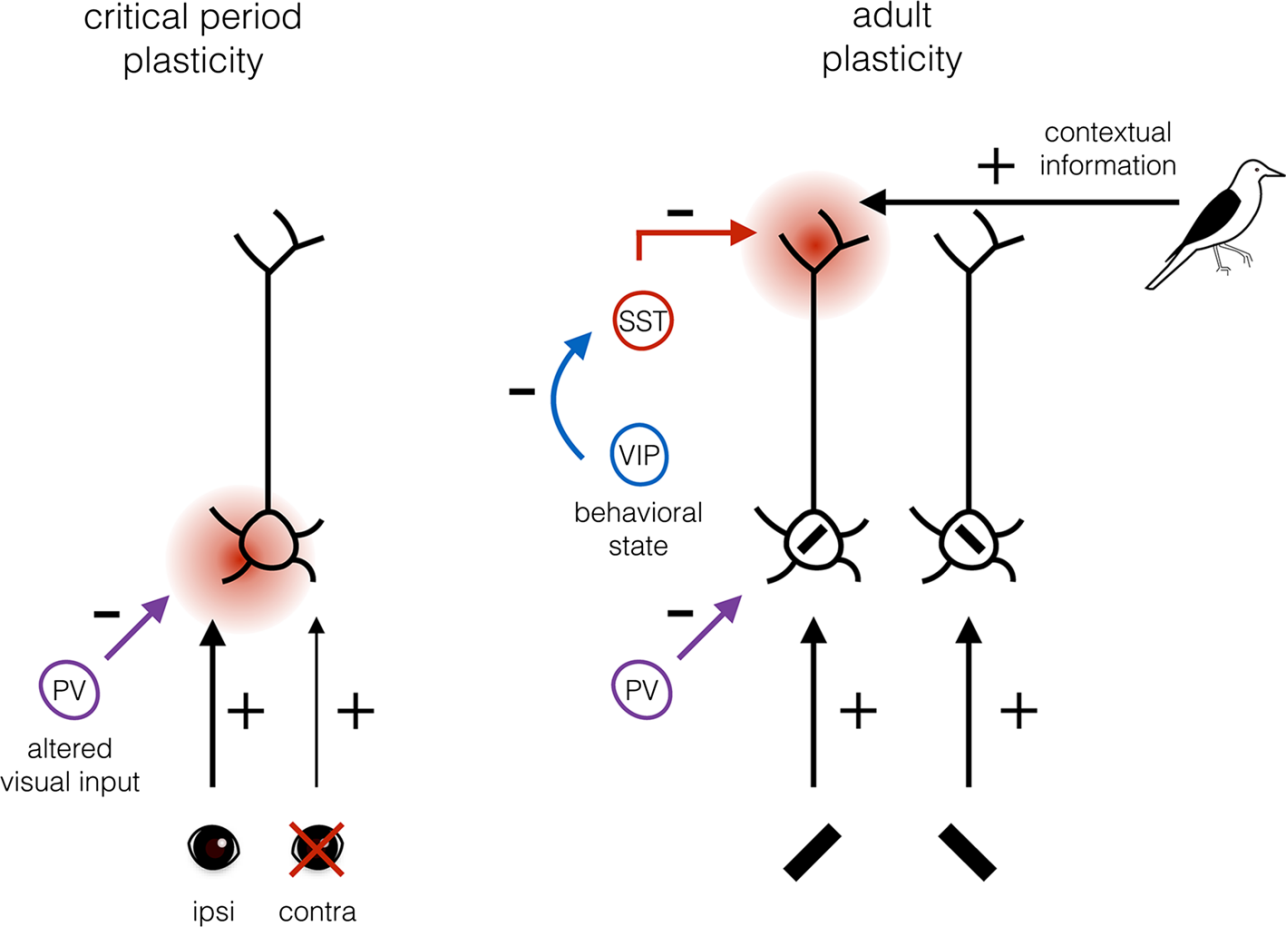
Proposed model of plasticity substrate selection by different interneuron subsets during the critical period and in adulthood. When visual input is altered by monocular deprivation during the critical period, net inhibition provided by PV+ interneurons decreases, so that feedforward connections can undergo plastic changes (indicated by the *red spot*), which is sufficient for learning. In the adult visual system, perceptual learning is reinforcement dependent and may involve plasticity of feedback connections providing contextual information about the feedforward inputs that are reinforced (in this example, the *black bar* with the retinotopy and orientation matching the bird’s black wing). This plasticity is facilitated by reduced inhibition of SST+ interneurons that innervate the dendritic tufts. Suppression of SST+ interneuron activity is mediated through inhibition by VIP+ interneurons whose activity depends on the behavioral state of the animal

Switching to visually driven neuronal activity as the substrate of plasticity may require the active suppression of spontaneous activity in V1 [106–108] (Fig. 3). During the critical period, PV+ interneurons have been shown to decrease spontaneous activity while leaving visual response strength unchanged [85], possibly favoring visual inputs over spontaneous activity as the substrate of cortical plasticity.

Furthermore, PV+ basket cell-mediated inhibition can control the timing precision of neuronal responses. Increasing their influence reduces the time window of temporal integration and spike-timing-dependent plasticity [76, 109]. The slowly progressing rise in inhibition during the critical period may thus gradually increase the stringency of plasticity and the temporal resolution of cortical activity in V1 while at the same time suppressing spontaneous activity and weak inputs. This eventually results in a stable, well-tuned, and fast network with limited noise.

## Regulation of PV+-basket-cell-mediated inhibition is crucial for critical period plasticity

Interestingly, PV+-basket-cell-mediated inhibition does not simply increase during the critical period, but is strongly influenced by visual input. Like excitatory neurons, they shift their ocular preference upon monocular deprivation [85, 110–112]. Furthermore, PV+ interneurons become temporarily suppressed upon a brief period of MD [47]. This rapid downregulation of PV+ interneuron activity is essential for inducing OD plasticity and disappears with critical period closure [47]. It has been suggested that plasticity of interneurons may cause selective suppression of deprived eye responses after MD [110, 113–115]. However, optogenetic reduction of PV+-, SST+-, or VIP+-interneuron-mediated inhibition after induction of OD plasticity does not cause any recovery of the OD shift, implying that such an instructive role of inhibition is improbable [85]. More likely, the temporary suppression of PV+ interneurons upon MD is essential for disinhibiting weak inputs from the open eye and widening the time window for synaptic integration. This reduction in the stringency of plasticity may help to recruit and strengthen new synaptic inputs after MD, allowing reoptimization of visual processing in V1.

As mentioned earlier, critical period closure can be interfered with by inactivating specific signaling cascades involving extracellular matrix- or myelin-based factors limiting axon growth. Recent studies show that inactivating some of these signaling cascades specifically in PV+ interneurons is sufficient to interfere with critical period closure [36, 39, 116–118]. This suggests that critical period closure involves mechanisms intrinsic to PV+ interneurons that limit their potential to temporarily reduce their activity. This idea is also supported by the finding that transplantation of immature interneurons into V1 enhances plasticity in adult mice [119–121].

Taken together, the function of PV+ basket cells in regulating the dynamic range and gating feedforward inputs may contribute to selecting visually driven inputs for cortical plasticity (Fig. 3). The control of PV+ basket cells over the window of temporal integration of synaptic inputs could at the same time define the timing on which the plasticity is based. Because the responses of PV+ basket cells are adjustable during the critical period, the stringency of these plasticity rules can be altered. This allows for the rewiring of V1 connectivity based on changes in visual input as long as the critical period lasts.

## Plasticity during adulthood

With the decline of critical period plasticity, there is an overall change in the main substrate of cortical plasticity. While during the critical period, feedforward connections, such as the thalamocortical projections, undergo extensive rearrangements, most types of plasticity that take place during adolescence and adulthood typically involve horizontal and feedback connections in V1. Their synapses are predominantly formed on distal dendrites and dendritic tufts in layer 1. These dendritic compartments are strongly innervated by SST+ interneurons and layer 1 NGF cells, which may underlie their dominant role in regulating plasticity during adulthood.

Various forms of plasticity can be induced in adult V1. These include adult ocular dominance plasticity, retinal-lesion-induced plasticity, and perceptual learning. Another type of adult plasticity in rodent V1 is stimulus-selective response plasticity. When a visual stimulus is presented repeatedly, V1 will become more responsive to this stimulus but not to others [122]. Surprisingly, this type of plasticity can result in eye-specific changes in cortical responsiveness and may well involve plasticity at thalamocortical connections [122]. In line with the idea that PV+ interneurons are involved in regulating plasticity of feedforward connections, stimulus-selective response plasticity has recently been found to involve changes in PV+-interneuron-mediated inhibition [123]. As little is known about the exact nature of stimulus-selective response plasticity and the excitatory and inhibitory connectivity that is involved [124], we will not discuss it further. However, the fact that it is induced by passive viewing and may alter feedforward connections means that the separation of the substrates of plasticity with age is not absolute.

## Plasticity induced by monocular deprivation or retinal lesions

Despite critical period closure, OD plasticity can still take place in the young adult cortex, though in a less efficient and permanent fashion than during the critical period [125–128]. In mice, a low level of OD plasticity can be induced up to 6 months of age [28]. While this phenomenon is particularly pronounced in mice, some OD plasticity after critical period closure is also observed in other species. In cats, for example, the critical period closes around 8 weeks of age, but some levels of OD plasticity can still be induced up to many months after birth [129]. Interestingly, OD plasticity in older cats does not involve layer 4, but is restricted to layers 2/3 and 5 [129]. A related form of cortical plasticity that can be readily induced in adult V1 across species is retinal-lesion-induced plasticity. Initially, V1 becomes unresponsive to the lesioned part of the retina. Over time, however, the lesion projection zone starts to respond to stimuli in neighboring visual-field positions [130–133]. This form of plasticity is also thought to involve the reorganization of horizontal connections in V1 [9]. Together, these findings support the idea that only during the critical period, feedforward connections undergo extensive plasticity, while horizontal and feedback connections are the main substrate of plasticity after critical period closure.

## Perceptual learning

One of the dominant types of plasticity that occurs in sensory systems after critical period closure is perceptual learning. It can be induced experimentally by instructing the subject, and/or by rewarding or punishing certain behaviors in response to a specific visual stimulus. Depending on the specific task, perceptual learning can result in changes in the responses of V1 neurons [30–34] as well as in higher visual areas [134, 135]. Studies in macaque monkeys, for example, have shown that when monkeys are taught to discriminate between visual stimuli with slightly different orientations, changes in orientation tuning occur in the pyramidal layers of V1. Interestingly, no such changes are observed in the input layer, layer 4 [33]. A similar observation has been made in mice learning an active avoidance task. Mice learned to initiate running on a treadmill when a visual stimulus of a defined orientation was presented. Failure to do so resulted in a mild shock. In these mice, anticipatory responses to the punishment could be recorded in layer 2/3 neurons of V1, but not in layer 4 [10]. It was also observed that in layer 4, neuronal responses became sparser. However, this also occurred when mice were not trained but passively viewed the same visual stimuli, suggesting some type of habituation occurred that was unrelated to perceptual learning. In some tasks, perceptual learning in primates has its strongest influence in V1 during task execution, which suggests that in these cases, alterations occurred in higher visual areas or in feedback connections from higher to lower areas, but not in the feedforward connections from the LGN to V1 [32]. Moreover, anesthesia, which suppresses feedback inputs, typically also suppresses learned changes in V1 responses [136]. Together, these findings illustrate that perceptual learning in V1 typically involves plasticity in the extragranular layers receiving feedback connections from other brain regions rather than the input layers receiving feedforward sensory information.

## Disinhibition during adult cortical plasticity

These forms of postcritical period plasticity are all associated with disinhibition. Chronic in vivo two-photon microscopy revealed that spines and boutons of interneurons are lost during retinal-lesion-induced plasticity in mouse V1 [50]. Other studies used gephyrin-GFP to label the postsynaptic side of inhibitory synapses. Using chronic in vivo imaging, these studies showed that inhibitory synapses formed onto pyramidal cell dendrites and spines in the top layers of V1 are rapidly eliminated when OD plasticity is induced in young adult mice [48, 49]. It is not yet clear what the identity is of the interneurons whose synapses are lost in these paradigms. Since volume release of GABA by NGF cells in layer 1 can strongly suppress the influence of callosal and possibly other layer 1 inputs on the dendritic tufts of layer 5 pyramidal cells [101], they are an interesting candidate.

There is more evidence suggesting that SST+ interneurons are the main cell type involved. For one, they form most of the inhibitory synapses in the top layers. In addition, it was recently found that in mice learning a motor task, inhibitory synapses on pyramidal neurons in motor cortex were also lost, specifically those formed by SST+ interneurons. Inhibitory synapses formed by PV+ interneurons persisted [137], although it should be mentioned that only PV+ boutons forming synapses close to the cell body were assessed, while it is known that PV+ basket cells also form inhibitory synapses on distal dendrites and even spines [138]. Enhancing or decreasing the activity of SST+ interneurons using optogenetics interfered with the learned behavior.

A more direct line of evidence suggesting the involvement of SST+ interneurons in regulating plasticity in adult V1 comes from studies analyzing the activity of SST+ interneurons during visual learning. In the previously mentioned active avoidance task in which mice learned to run in response to a visual stimulus to avoid a mild shock, it was observed that SST+ interneurons became less active. Increasing their activity interfered with the learned task [10]. Another series of studies found that when adult mice were running on a treadmill, while visual stimuli were presented, this resulted in the suppression of SST+ interneurons and a facilitation of OD plasticity [139, 140]. Others did not find evidence for SST+ interneuron suppression during running [141], and the cause of this apparent discrepancy still needs to be resolved. This notwithstanding, virally mediated expression of tetanus toxin in SST+ interneurons, which suppressed GABA release, also enhanced adult OD plasticity [14]. Together, these studies suggest that release from SST+-interneuron-mediated inhibition enhances adult plasticity.

It thus appears that the change in the substrate of plasticity matches the interneuron subsets involved in regulating plasticity. While PV+ interneurons gating feedforward inputs regulate critical period plasticity, SST+ interneurons forming most inhibitory synapses in layer 1 and gating horizontal and feedback connections appear to be important regulators of adult cortical plasticity (Fig. 3).

## Disinhibitory circuits

How can reduction of SST+-interneuron-mediated inhibition be achieved specifically during learning? Connectivity studies have found that SST+ Martinotti cells are innervated by VIP+ interneurons [87, 90, 92]. VIP+ interneurons, in turn, are extensively innervated by neurons in other brain areas [140] and express nicotinic acetylcholine receptors and serotonin receptors making them highly sensitive to neuromodulatory inputs [58, 142]. Through these long-range connections, VIP+ interneurons can thus be activated during behavioral states in which learning is required. In various brain regions of the mouse, VIP+-interneuron-mediated disinhibition has been found to involve modulatory inputs that signal reward, punishment, or arousal [92, 93, 143], consequently suppressing SST+ interneuron activity and releasing inhibition of horizontal or feedback connections. This disinhibition, in turn, stimulates plasticity. Similarly, in V1, the enhancement of adult OD plasticity in mice running on a treadmill required the activation of VIP+ interneurons [140]. Optogenetically activating VIP+ interneurons also enhanced adult OD plasticity even when the mice did not run [14].

How reducing SST+ interneuron mediated inhibition may enhance plasticity is under intense investigation. It is, however, tempting to speculate that disinhibition permits the potentiation of relevant feedback connections: those that provide the contextual information about feedforward inputs, which is relevant for making a choice leading to reward. To illustrate this, imagine a situation in which a subject needs to learn to recognize the image of a particular bird (Fig. 3). Every time the image of this bird is shown, and the subject can correctly differentiate it from other bird images; a reward is given. Neurons in V1 will respond to the image of the bird. However, feedforward inputs to a particular neuron in V1 could be just the same when a picture of another bird is shown. Only feedback connections to this neuron can provide contextual inputs differentiating between the various images. When these contextual inputs are disinhibited through reinforcement signals, their inputs may become strengthened causing the neuron in V1 to become more responsive to the feedforward input, but only when it is presented as part of the rewarded bird image. This may significantly improve the efficiency by which the image is recognized.

An important question is whether disinhibition is in any way specific for the feedforward or feedback inputs that are being reinforced. For example, different VIP+ interneurons may be selectively activated by different contextual- or behavioral state-dependent inputs. Some may be activated by running, others by reward, punishment, or specific contextual feedback signals. Moreover, SST+ interneurons may be tuned to the feedforward inputs that are reinforced, or selectively innervate dendritic branches or spines that receive relevant contextual inputs. Evidence for these conditions has been found. For example, stimulus-specific disinhibition has been observed in V1, mediated through long-range connections from cingulate cortex onto VIP+ interneurons [144]. It is also known that SST+ interneurons have well-defined receptive fields, and show orientation tuning [72, 85, 144, 145]. Interestingly, SST+ interneurons often form inhibitory synapses onto dendritic spines [49, 146]. This wiring allows for the selective inhibition, and thus also disinhibition, of synaptic inputs. In support of such selective disinhibition, it was recently shown that when mice learn two different motor tasks, different dendritic branches of layer 5 pyramidal neurons show Ca^2+^ spikes [15]. Silencing SST+ interneurons caused a loss in the branch specificity of these Ca^2+^ spikes. SST+ interneuron silencing did not reduce learning of a single task, but did cause decreased performance in a previously learned task, once a second task was learned. Thus, SST+ interneurons appear to gate specific inputs to dendritic tufts and their suppression by VIP+ interneurons may allow the strengthening of selective inputs relevant to the task to be learned (Fig. 3). Unraveling the connectivity rules between interneurons of different subtypes and with different selectivity for visual or behavioral stimuli will be crucial for understanding how cortical plasticity is regulated, but may turn out to be a daunting task.

## Conclusions and future directions

In summary, during different stages of brain maturation, plasticity is boosted by temporary disinhibition. However, the interneurons involved, the underlying mechanisms, and the inputs that undergo plasticity differ depending on the developmental stage and condition under which plasticity occurs. What is the use of this temporary disinhibition? We hypothesize that information processing is more efficient when signal-to-noise ratios are high. However, this comes at the cost of suppressing information that may be essential to execute tasks that are not routine and require learning. Thus, the downregulation of interneuron activity under the right circumstances may help to serve these opposing needs.

Many fundamental questions remain to be answered. During development, critical periods in different brain areas occur at different stages of postnatal development. Generally speaking, higher cortical areas undergo plasticity at a later stage than lower areas. An important question is how this timing is regulated. The timing of the critical period in V1 is partially regulated through retinal input, which drives the development of the extracellular matrix and inhibitory innervation [147]. Do high cortical areas wait for a particular type of input from lower areas? Or is a strict genetic program followed? It is also unknown to what extent the development of feedback connections awaits closure of the critical period. It would be especially important to understand whether inhibitory inputs in layer 1 hold off the development or plasticity of these feedback inputs. This could be regulated through an initially strong influence of NGF cells on dendritic tufts or low influence of VIP+ interneurons over SST+ interneurons during the critical period. However, until now, it remains unknown what role VIP+-, SST+- or NGF interneurons play during the critical period. Similarly, it has been noticed that after the critical period, OD plasticity can only be induced in young adult but not in older mice [28]. Possibly, SST+ interneurons may also become less controllable with age, thus further reducing plasticity of horizontal connections. This may explain why transplantation of embryonic SST+ interneurons enhances adult OD plasticity [121].

It remains unclear whether the connectivity of different interneuron subsets as described in V1 is the same in other cortical areas. Studies on disinhibition in auditory, sensory, visual, prefrontal, and motor cortices have already provided some apparently contradicting results [87, 92, 93, 143]. Most likely, general connectivity rules between interneuron subtypes exist across the neocortex. However, at the same time, many different subtypes of SST+, PV+, VIP+, and NGF interneurons may exist with diverse connectivity patterns and properties. These patterns may well be dependent on the function of the cortical area and the specific responsiveness of interneurons to various behavioral conditions and sensory inputs. The identification of additional genetic markers to further subdivide the various interneuron populations may help understanding the connectivity rules of cortical inhibition [100]. In addition, extensive connectivity studies [87, 90, 98, 148] of interneurons whose functional properties have been determined in vivo, as done for excitatory neurons, [149] will be required for solving this complex puzzle.

Interneuron dysfunction has been implicated in many neurodevelopmental disorders, including autism, schizophrenia, and intellectual disability [150, 151]. The increasing knowledge on the role of inhibition in the regulation of critical periods during development and reinforcement learning later on is likely to open up new avenues to treat these disorders. This may involve extending or reactivating critical periods to correct or prevent maladaptation of the developing networks, or altering inhibitory tone to improve the learning ability of people suffering from these disorders [152]. In rodents, several approaches that alter cortical inhibition have proved effective in increasing plasticity in V1, including environmental enrichment [153, 154], housing animals in the dark [155, 156], degrading the extracellular matrix [39], treatment with serotonin reuptake inhibitors [157], and opto- or pharmacogenetically altering interneuron activity [14, 47]. To develop selective and powerful approaches to enhance cortical plasticity in human patients, it is crucial that we identify the exact working mechanisms and targets of these treatments. A better understanding of how inhibition and disinhibition regulate cortical plasticity is, therefore, indispensable.
